# Features of Nonsuicidal Self-Injury and Relationships with Coping Methods among College Students

**Published:** 2019-02

**Authors:** Jinting WU, Hairong LIU

**Affiliations:** 1. Second Affiliated Hospital of Wannan Medical College, Wuhu, 241002, China; 2. Department of Public Administration, Wannan Medical College, Wuhu, 241001, China

**Keywords:** Nonsuicidal self-injury, Influential factors, Correlation analysis

## Abstract

**Background:**

This study investigated the nonsuicidal self-injury (NSSI) presentation and the features of coping methods among college students, in addition to analyzing the factors that influence self-injury behavior.

**Methods:**

From April to November 2016, 2,520 undergraduate students who were studying in some colleges in Anhui Province in China were surveyed using the Self-Injury Behavioral Survey Questionnaire and the Coping Styles Questionnaire (CSQ).

**Results:**

The detection rate of self-injury behavior among college students was 13.73%. Frequent Internet use and smoking were associated with self-injury behavior (*P* < 0.05). There were statistically significant differences between the self-injury group and the non-self-injury group in terms of the coping methods of problem-solving, self-reproach, help-seeking, and illusions (*P* < 0.01). According to the logistic regression analysis of the students, their place of origin, mother’s education, family finances, family type, family relationships, gender, frequent Internet use, and smoking were associated with self-injury behavior (*P*< 0.01). Self-reproach and withdrawal were risk factors for self-injury behavior while problem-solving and rationalization were protective factors for self-injury behavior.

**Conclusion:**

Students who frequently use the Internet and smoke are more prone to self-injury. College students’ choices of problem-solving and rational coping styles in the face of pressure are conducive to preventing nonsuicidal self-injury.

## Introduction

Nonsuicidal self-injury (NSSI) primarily refers to self-injury behaviors without suicidal intent; these are actions of intentional self-harm people take after experiencing intense emotional pain, with the goal of releasing negative emotions or alleviating interpersonal conflicts (1,2). Unlike suicide, the goal of NSSI is not to end life; the vast majority of individuals who engage in NSSI do not seek clinical attention and thus tend to be over-looked. Although individuals engaged in NSSI do not aim to commit suicide, they can cause serious harm to their mental and physical health due to repeated self-injury and are at a greater risk of suicide than those not engaged in NSSI. NSSI is commonly found among adolescents and may last many years. Related studies have shown that NSSI has become a key factor that threatens the mental and physical health of adolescents (3). An epidemiological study of American adolescents by Taliaferro et al. found that the detection rate of NSSI was 7.3% (4). Hamza et al. found an NSSI detection rate of 14.9% among German adolescents (5). A study of Japanese college students by Tresno et al. found an NSSI detection rate of 10% (6). It seems, therefore, that NSSI among adolescents has become a prevalent social and public health problem worldwide. In the past, NSSI was considered a symptom of borderline personality disorder. However, an increasing number of scholars are inclined to see NSSI as an independent clinical disorder. The DSM-V included it in its appendix and proposed referential diagnostic criteria, calling for additional research (7).

Research on NSSI is in the preliminary stages, and its physiological and psychological pathology are not yet clear. The DSM-V introduced two theories based on functional behavioral analysis. The first is based on learning theory and stresses the effects of positive and negative reinforcement on behavior. The second sees NSSI as a means of self-punishment, a behavior to make up for causing pain or injury to other people. Based on studies from various locations, the influential factors related to NSSI include environmental factors as well as individual psychological and neurobiological factors. External environmental stimuli produce emotions and behaviors after an individual’s cognitive assessment; NSSI is thus an individual’s response to external stimuli. The integrated model (8) regards NSSI as a coping mechanism or a means of communication; through self-injury behavior, the person realizes self-control and self-management, and uses this method to affect or control others. Therefore, we used an important modulating factor in the psychological stress process—coping methods—to explore the influential factors of NSSI. Coping methods affect behaviors and emotions as the mediating mechanism between health and stress, and are cognitive and behavioral methods individuals adopt when faced with setbacks and pressure. College students, between late adolescence and early adulthood, are in an important stage of development. They are the key targets of talent cultivation by various governments, as well as a group at high risk of NSSI. Therefore, governments focus on college students’ health problems not only as important quality-of-life issues but also as a way to ensure human resources for sustainable development. It is thus necessary in a practical sense to study NSSI in college students.

This study analyzed the features of coping methods and NSSI epidemiological traits among college students to explore the influential factors of self-injury behavior. It is hoped that the findings will further enrich theoretical research on NSSI and provide a basis for preventing NSSI and maintaining the health of adolescents.

## Materials and Methods

### Research subjects

Stratified cluster sampling survey by grade was administered to college students in Anhui Province in China. A total of 2,520 questionnaires were administered, and 2,448 valid questionnaires were retrieved. There were 1,272 male subjects and 1,176 female subjects, aged 20.21 ± 0.94 years.

The investigation was based on the principle of voluntary and informed consent. This study was approved by the Ethics Committee of Second Affiliated Hospital of Wannan Medical College.

### Research methods

#### Coping Strategies Questionnaire

The Coping Styles Questionnaire (CSQ) includes 62 questions, divided into 6 subscales: problem-solving, self-reproach, help-seeking, illusions, withdrawal, and rationalization. Each subscale is made up of relevant questions, and each question has only two possible answers: “yes” and “no.” The responses are used to understand the types of coping methods and features of coping behaviors used by individuals or groups (9). We used the questionnaire to analyze different types of coping methods.

#### Self-Injury Behavioral Survey Questionnaire

A Self-Injury Behavioral Survey Questionnaire was formulated in conjunction with existing survey questionnaires and relevant survey results. It mainly covers general data and injury conditions, with 20 questions on self-injury behavior, which were used to understand the subject’s self-injury behavior and whether suicidal thoughts had been present in the last six months. Self-injury was evaluated in terms of frequency and severity. Frequency was divided into 0 times, 1 time, 2–4 times, and more than 5 times. Severity was divided into 4 types: none, mild, moderate, and severe. Of these, “none” referred to no damage to the skin, “mild” referred to mild injury to the body, “moderate” referred to some damage that could be treated by the individual, and “severe” referred to severe injury requiring hospitalization.

#### Data processing and statistics

We conducted group testing and used SPSS 21.0 to conduct statistical analyses of all data. Quantitative data were presented as *x̄*±*s*, and an X-test or Fisher’s exact probability method was used to compare multiple groups of qualitative data. Logistic regression was used to explore the relationship between possible influencing factors and various self-injuring behaviors. The enter method was used to screen variables, and the criterion for selecting variables for single-factor logistic regression was *P* < 0.10. The inspection level was 0.05.

## Results

### General features

A total of 2,520 college students completed the survey, and 2,448 questionnaires were valid and accounted for. Among them, 2,112 did not report engaging in NSSI (hereafter, the “non-self-injury group”) while 336 had engaged in NSSI (hereafter, the “self-injury group”), for a detection rate of 13.73%; 6.62% were male (162 students), and 7.11% were female (174 students).

### Population distribution of self-injury behavior

Statistical analysis of the population distribution of the self-injury group included analyses in terms of gender, family background, individual daily behaviors, and understanding of self-injury. There were no significant differences in the detection rates of self-injury behavior between men and women (*P* > 0.05). However, students who frequently used the Internet and those who frequently smoked had significantly higher rates of self-injury behavior than those who did not (*P* < 0.01). In addition, students who believed self-injury was preventable had lower detection rates of self-injury behavior (*P* < 0.05) (Table 1).

**Table 1:** Comparison of self-injury behavior among different groups

***Population feature***	***Persons surveyed***	***Incidence of self-injury behavior, N (%)***	***χ^2^***	***P***
**Gender**			2.190	0.139
Male	1,272	162 (12.74%)		
Female	1,176	174 (14.80%)		
**Place of origin**			0.233	0.629
Rural	1,035	138 (13.33%)		
Urban	1,413	198 (14.01%)		
**Father’s education**			47.97	0.000
Elementary school or less	414	72 (17.39%)		
Middle school	1,098	168 (15.30%)		
High school or secondary school	534	84 (15.73%)		
College or higher	402	12 (2.99%)		
**Mother’s education**			22.27	0.000
Elementary school or less	816	144 (17.65%)		
Middle school	882	120 (13.61%)		
High school or secondary school	708	70 (10.03%)		
College or higher	42	2 (4.76%)		
**Family finances**			15.50	0.001
< RMB 1,000	174	28 (16.09%)		
RMB 1,000–3,000	402	72 (17.91%)		
RMB 3,000–5,000	1,086	156 (14.36%)		
> RMB 5,000	786	80 (10.18%)		
**Family type**			33.92	0.000
Nuclear family	1,836	222 (12.09%)		
Extended family	528	108 (20.45%)		
Single-parent family	54	0 (0)		
Reconstituted family	30	6 (20.00%)		
**Family relationships**			14.08	0.0002
Harmonious	2,196	282 (12.84%)		
Unharmonious	252	54 (21.43%)		
**Often uses the internet**			33.91	0.000
Yes	720	144 (20.00%)		
No	1,728	192 (11.11%)		
**Often smokes**			41.63	0.000
Yes	78	30 (38.46%)		
No	2370	306 (12.91%)		
**Has had safety education**			26.27	0.000
Yes	2,202	276 (12.53%)		
No	246	60 (24.39%)		
**Only child**			11.86	0.0006
Yes	1,317	210 (15.95%)		
No	1,131	126 (11.14%)		
**Believes that injury is preventable**			19.00	0.000
Yes	1,404	156 (11.11%)		
No	1,044	180 (17.24%)		

Note: “Often smokes” was defined as more than 5 cigarettes/day, at least 20 a month, lasting more than 6 months. “Often uses the Internet” was defined as 4 or more consecutive hours spent online each day on average for more than 3 months

### Multiple logistic regression analysis

To analyze the factors affecting self-injury behavior among college students, the occurrence of self-injury behavior was set as the dependent variable, where 1 = self-injury group and 0 = non-self-injury group. The factors of gender, frequent smoking, frequent Internet use, parents’ education, and family finances were set as the independent variables. Based on an inclusion criterion of 0.05 and an exclusion criterion of 0.10 for the independent variables, multivariate unconditional logistic regression analysis was performed (stepwise regression, Table 2). Table 2 shows that in terms of the rearing environment of college students, their place of origin, mother’s education, family finances, family type, and family relationships were correlated with self-injury behavior.

**Table 2:** Multivariate unconditional logistic regression analysis of influential factors in self-injury behavior

***Variance***	***β***	***Wald x^2^***	***P***	***OR***	***95% CI***
Gender	0.459	11.150	0.001	1.583	1.209–2.073
Place of origin	0.551	13.676	0.000	1.734	1.295–2.322
Father’s education	−0.174	3.657	0.056	0.840	0.703–1.004
Mother’s education	−0.301	7.246	0.007	0.740	0.595–0.921
Family finances	−0.309	15.692	0.000	0.734	0.630–0.856
Family type	0.195	3.935	0.047	1.216	1.002–1.474
Family relationships	0.656	12.778	0.000	1.928	1.345–2.763
Often uses the internet	−0.393	9.107	0.003	0.675	0.523–0.871
Often smokes	−2.671	105.424	0.000	0.069	0.042–0.115
Educated	−0.204	0.746	0.388	0.815	0.513–1.295
Only child	−0.011	0.006	0.937	0.989	0.749–1.306
Believes that injury is preventable	0.160	1.421	0.233	1.173	0.902–1.526
Constant	3.291	19.072	0.000	26.876	

This indicated that an urban origin, low level of education by the mother, low family income, single-parent or reconstituted families, and unharmonious family relationships were risk factors for self-injury behavior. In terms of college students’ personal traits, gender, frequent Internet use, and smoking were correlated with self-injury behavior. Female students were more likely than males to engage in self-injury behavior (OR = 1.583, 95% CI: 1.209–2.073). College students who frequently used the Internet or smoked were more likely than those who did not to engage in self-injury behavior (OR = 0.675, 95% CI: 0.523–0.871; OR = 0.069, 95% CI: 0.042–0.115).

### Effect of coping methods on self-injury behavior

Analysis of the traits of coping methods among the surveyed college students was conducted by comparing the coping methods of the self-injury group and the non-self-injury group. There were statistically significant differences in terms of the factors of problem-solving, self-reproach, help-seeking, and illusions. Compared to the non-self-injury group, college students who engaged self-injury behavior used problem-solving and help-seeking as coping methods relatively less (*P* < 0.01) and were more inclined to use self-reproach and illusions as coping methods (*P* < 0.01) (Table 3).

**Table 3:** Comparison of coping methods between college students in the self-injury and non-self-injury groups

***Coping method***	***Groups***		***t***	***P***
	***Non-self-injury group (2,112)***	***Self-injury group (336)***		
Problem solving	0.848 ± 0.164	0.731 ± 0.216	11.519	0.000
Rationalization	0.380 ± 0.197	0.373 ± 0.208	0.563	0.573
Self-reproach	0.242 ± 0.261	0.357 ± 0.301	7.332	0.000
Help seeking	0.661 ± 0.225	0.586 ± 0.230	5.637	0.000
Illusions	0.384 ± 0.238	0.425 ± 0.248	2.951	0.003
Withdrawal	0.370 ± 0.237	0.384 ± 0.258	0.996	0.319

To analyze the effects of coping methods on self-injury behavior, self-injury behavior was set as the dependent variable, where 1 = the self-injury group and 0 = the non-self-injury group. The various factors of the coping methods were set as independent variables, based on the inclusion criteria and using multivariate unconditional logistic regression analysis.

The results showed that among the factors of coping methods, self-reproach and withdrawal were the risk factors for self-injury behavior (OR = 14.961, 95% CI: 7.177–31.187; OR = 0.283, 95% CI: 0.115–0.699) while problem-solving and rationalization had the opposite effect on self-injury behavior, functioning as protective factors (OR = 0.088, 95% CI: 0.046–0.167; OR = 0.243, 95% CI: 0.089–0.664) (Table 4).

**Table 4:** Multivariate unconditional logistic regression analysis of coping methods’ effects on self-injury behavior

***Variance***	***β***	***Wald x^2^***	***P***	***OR***	***95% CI***
Problem solving	−2.431	54.977	0.000	0.088	0.046–0.167
Rationalization	−1.414	7.615	0.006	0.243	0.089–0.664
Self-reproach	2.705	52.113	0.000	14.961	7.177–31.187
Help seeking	−0.015	0.003	0.960	0.985	0.559–1.737
Illusions	0.089	0.043	0.836	1.093	0.470–2.545
Withdrawal	1.262	7.501	0.006	0.283	0.115–0.699
Constant	0.282	1.199	0.274	1.326	

## Discussion

In recent years, the detection rates of NSSI have been inconsistent. Collectively, research has indicated that the incidence of self-injury behavior among college students is relatively high. Studies have found the incidences in Changsha, China, and Taiwan to be 10.66% and 23%, respectively (10,11). The incidence rate of self-injury behavior was 26.8% and was significantly higher among men than women (12). Wang et al. (12) found an incidence of self-injury behavior among college students of 15.1% (13) while Duan et al. found a rate of 30.72% (14). The present study surveyed current college students and found an NSSI incidence rate of 13.73%, without significant differences between men and women. This detection rate is lower than the rates found in recent studies. Such differences in the detection rates of self-injury behavior could be attributable to different research objectives, inconsistent survey questionnaires, and different levels of cultural acknowledgment of self-injury behavior. In addition, compared to other domestic studies of college students, some subjects in this study were medical students. Given their medical focus, they receive more health education, which might also facilitate the prevention of self-injury behavior.

The causes of NSSI are relatively complex. Generally, they can be divided into extrinsic and intrinsic (self-related) factors. This study used an epidemiological survey to analyze the influential factors of NSSI. Studies have shown that adolescent emotions and behaviors are affected to a significant degree by family function, family structure, and interactive relationships among family members; family finances might also be a risk factor for self-injury behavior (15–17). Through logistic regression analysis, this study found that in terms of extrinsic factors, college students’ place of origin, mother’s education, family finances, family type, and family relationships were correlated with self-injury behavior. College students from urban areas, with low maternal education, low family income, single-parent or reconstituted families, and unharmonious family relationships were more likely to exhibit self-injury behavior. This demonstrates the importance of mothers and family atmosphere in children’s developmental education. Low education on the part of the mother and an unharmonious family atmosphere tend to complicate child-rearing, affecting children’s cognitive development or even directly causing psychological trauma that induces self-injury behavior. Meanwhile, students who grow up in cities may lack sufficiently mature coping methods when faced with stress due to relatively privileged backgrounds, making them more passive. A low family income may cause students to face more difficulties in their daily lives. In our study, we understood that aside from academics, students from difficult backgrounds also have to consider family burdens. They often engage in frugal work-study or part-time work on weekends and holidays to alleviate economic pressure. In terms of intrinsic factors, this study found that certain behaviors among college students were related to NSSI. Students who frequently used the Internet or smoked were more likely to exhibit self-injury behavior. This could be because frequent Internet use and smoking tend to suggest that students are experiencing high internal stress, a lack of direction, or low self-demand. Receiving preventative and safety education is beneficial for preventing self-injury behavior. Thus, one effective way to prevent self-injury behavior is to actively develop effective safety education. In addition, our study found that female college students were more likely to engage in NSSI than male college students, which is consistent with most prior research (18). A few studies, however, found a higher rate of self-injury behavior in men than women (19). This could be related to the categories of self-injury behavior examined in those studies and warrants further investigation.

Behaviors are the outcomes of an individual’s response to the external environment through cognitive assessment. Different cognitive styles affect an individual’s behavioral choices. By focusing on the features of coping methods among college students, this study elucidated how different coping methods affect self-injury behavior. The data showed that the rate of self-injury behavior was lower among students who believed injury was preventable; thus, cognitive assessment regarding injury affects self-injury behavior. In the analysis of the features of college students’ coping methods, the scores of the coping method factors were ranked, from high to low, as follows: problem-solving, help-seeking, illusions, rationalization, withdrawal, and self-reproach. Coping methods tended to be mature and mixed. In the study, through an analysis of the self-injury group’s coping methods, we found that the self-injury group and the non-self-injury group differed significantly in terms of problem-solving, self-reproach, help-seeking, and illusions. This indicated that there was a correlation between the different coping methods adopted by college students and the occurrence of NSSI. Through multivariate unconditional logistic regression analysis, we discovered that self-reproach and withdrawal were risk factors for the occurrence of NSSI while problem-solving and rationalization were protective factors. The more a student was inclined to choose immature coping methods, such as self-reproach and withdrawal, the greater the likelihood of NSSI. Conversely, the more a student was inclined to choose coping methods, such as problem-solving and rationalization, the lower the likelihood of NSSI.

This could be because problem-solving is a mature coping method; thus, students employing it could rationally understand and face stress, thereby rationalizing extrinsic stress through internalization to proactively deal with difficulties. Conversely, self-reproach and withdrawal are immature coping methods that may convert extrinsic stress into intrinsic psychological stress. Students employing these methods tended to blame themselves when faced with difficulties, which in turn produced passive responses, resulting in self-injury behavior to punish themselves in order to achieve inner balance. This is consistent with the emotional management function assumptions for self-injury behavior (20).

## Conclusion

This study examined NSSI among college students from the perspective of coping methods. It accounted for NSSI as an individual response strategy to explore the factors that affect NSSI based on internal and external factors in order to analyze how coping methods affect NSSI. Gender, place of origin, mother’s education, family finances, family type, and family relationships were correlated with self-injury behavior. Frequent Internet use and smoking were associated with self-injury. Choosing problem-solving and rational coping styles in the face of pressure is conducive to preventing NSSI. Our findings highlight the need to actively emphasize students’ personal traits in conjunction with their lifestyles and family backgrounds to develop active and healthy coping methods. This could help to effectively reduce the occurrence of self-injury behavior.

## Ethical considerations

Ethical issues (Including plagiarism, informed consent, misconduct, data fabrication and/or falsification, double publication and/or submission, redundancy, etc.) have been completely observed by the authors.
